# Application of Poultry Gelatin to Enhance the Physicochemical, Mechanical, and Rheological Properties of Fish Gelatin as Alternative Mammalian Gelatin Films for Food Packaging

**DOI:** 10.3390/foods12030670

**Published:** 2023-02-03

**Authors:** Azam Ashrafi, Hamid Babapour, Simindokht Johari, Faezeh Alimohammadi, Farangis Teymori, Abdorreza Mohammadi Nafchi, Nurul Nuraliya Shahrai, Nurul Huda, Ahmadreza Abedinia

**Affiliations:** 1 Department of Food Science and Technology, Science and Research Branch, Islamic Azad University, Tehran 14778-93855, Iran; 2 Food Biopolymer Research Group, Food Science and Technology Department, Damghan Branch, Islamic Azad University, Damghan 36716-39998, Iran; 3 Department of Food Science and Technology, Azadshahr Branch, Islamic Azad University, Azadshahr 89985-49617, Iran; 4 Food Technology Division, School of Industrial Technology, Universiti Sains Malaysia, Minden 11800, Penang, Malaysia; 5 Faculty of Sustainable Agriculture, Universiti Malaysia Sabah, Sandakan 90509, Sabah, Malaysia; 6 Department of Food Engineering, Inonu University, Malatya 44280, Turkey

**Keywords:** gelatin, modification, film properties, rheological properties, tensile strength, WVP, sachet, olive oil, peroxide index

## Abstract

This study aimed to describe the properties of cold water fish gelatin (FG) blended with poultry gelatin (PG) for a production of a sachet containing olive oil. To find a desirable film, the different ratio of FG-PG-based films were characterized in terms of mechanical properties. As the proportion of PG in PG-FG-based increased, the tensile strength and Young’s modulus were increased, and the elongation at break and heat seal strength of the films were decreased. The 50-50 film had favorable characteristics to use as a sachet. The amount of acid index and peroxide of the oil stored in the sachets after 14 days showed that there is a significant difference (*p* < 0.05) between the films. The barrier properties of the films including the water vapor permeability and oxygen permeability of films were increased from 1.21 to 4.95 × 10^−11^ g m^−1^ Pa^−1^ s^−1^ and 48 to 97 cm^3^ mµ/m^2^ d kPa, respectively. Dark, red, yellow, and opaque films were realized with increasing PG. Fourier transform infrared (FTIR) spectra approved a wide peak of approximately 2500 cm^−1^. The rheological analysis indicated that, by adding PG, viscosity, elastic modulus (G′) and loss modulus (G′′) were increased significantly (*p* < 0.05) about 9.5, 9.32 and 18 times, respectively. Therefore, an easy modification of FG with PG will make it suitable for oil sachet packaging applications for the food industry.

## 1. Introduction

Mammalian gelatins (MG) are rapidly becoming a key tool in the pharmaceutical, food and cosmetic industries due to their physicochemical and rheological properties and desirable film formation. These types of gelatins are traditionally derived from bone and connective tissue of cattle and pork species [1]. However, its application has been limited due to health-related concerns and religious constraints, which contributes to the risk of prions transmission. Therefore, the last decade has seen a growing trend toward global gelatin consumption in various industries annually. In this regard, other sources such as marine and poultry were introduced [1,2]. General health, religious and cultural concerns about the consumption of products containing gelatin can be stated as the following [2], which is why scientists are interested in identifying viable alternative sources for extracting gelatin from non-mammalian sources. Judaism and Islam forbid the consumption of pigs, while Hinduism forbids the consumption of beef products. On one hand, the halal issue in Islam raises questions about how to find kosher sources for the Muslim, Jewish and Hindu populations of the world in the next 20 years (with a projected population increase rate of 53 percent, reaching about 3.7 billion people), and on the other hand, costly and time-consuming techniques such as chromatography, chemical absorption, mass spectrometry, etc. to determine the origin of gelatin species.

Cold-water fish gelatins have low rheological properties such as lowering the melting and gelation temperatures and reduced gel strength due to their lower content of imino acid (proline + hydroxyproline) compared to MG. However, the gelatins extracted from the skins of warm-water fish species (tilapia, tuna, black carp) are somewhat similar in content to these amino acids in the skin of pigs and cows [1]. Cold-water fish gelatins have a low gelling point ranging from 4 to 12 °C, bloom or gel strength (220 to 600 g), and a melting point (11 to 21 °C), respectively, although these amounts are 20 to 28 and 20 to 27 °C (gelling point), 75 to 818 and 100 to 300 g (gel strength), and 27 to 42 and 28 to 31 °C (melting point) for PG and MG, respectively [1,2]. The low temperature of FG makes it suitable for producing complex coacervation at the above-ambient temperature [2,3], but as mentioned, this shortcoming limits its application in other cases. From the economic environmental point of view, FG derived from fish by-products such as skin, bone, scales and cartilage is valuable in the industry of fish processing [4,5].

On the other hand, the characteristic of FG for the ability to form a good film and its abundance in nature has introduced it as a preferred raw material in the production of biodegradable packaging [6]. However, many studies have been conducted to improve the properties of FG films, which include adding various compounds to FG, such as palm wax [7], palm oil [8], epigallocatechin gallate [9], haskap berries (*Lonicera caerulea* L.) extract [10], chitosan combined with ε-polylisin (ε-PL) [11], phycocyanin, hydroxy Tyrosol, 3,4-dihydroxyphenylglycol and anthocyanins, silver nanoparticles doped with titanium dioxide and chitosan nanoparticles containing polyphenols and agar [12].

Moreover, a number of research studies were focused on the modification of FG by enzymatic methods such as microbial-transglutaminase (MTGase) [13], chemical modification such as phosphorylation [14], aldehyde [15], phenolic and physical modification [4]. Although the above methods have been successfully used to modify FG to improve its gel and rheological properties, due to the high cost of enzymatic methods and the use or production of toxic reagents by chemical methods, they require complications and have some other disadvantages, such as the formation of polymers with high molecular weight and the drastic change in the structure of FG, so that the melting point and strength of FG gel increase significantly, which leads to the strength and hardness of the gel. It can even cause the loss of its thermal reversibility. The simplest and most widely used method to improve the properties of FG gel is physical modifications such as adding electrolytes and non-electrolyte salts or blending [1,4].

Poultry gelatin (PG) and FG are most of the suggested sources that have been studied as alternatives for MG. The main advantage of PG is stronger gel strength and comparable stable gel structure for MG, which has increased the hope for its widespread use. In contrast, the main disadvantage of FG, especially cold-water, is weaker gel strength and less stable gel structure than MG, which has limited its use [2]. Therefore, by adding PG to FG, its weak structure can be improved. According to the best of our information, higher bloom gelatin mixed methods have not been used to modify FG, and in this study, we used ratios of 0:100, 25:75, 50:50, 75:25, and 100:0 for PG-FG to select the best film to apply as olive oil packaging and evaluation of physicochemical of sachets during storage time of 14 days in room temperature.

## 2. Materials and Methods

### 2.1. Materials

Pekin duck feet were purchased from Perak Duck Food Industries Sdn. Bhd. (Penang, Malaysia). The cold-water fish gelatin (G7041) (FG) and type B bovine gelatin (BG) (G9382) as MG were purchased from Sigma Chemical Co. (St. Louis, MO, USA). Glycerol was obtained from Sigma-Aldrich, Steinheim, Germany. Olive oil was purchased from the local market. Acetic acid, ethyl alcohol, sodium hydroxide (NaOH), Magnesium nitrate (Mg (NO_3_)_2_), Phosphorus pentoxide (P_2_O_5_) and sodium thiosulfate were purchased at an analytical grade.

### 2.2. Preparation of Gelatin Specimens

The poultry gelatin (PG) was extracted from duck feet. The raw samples were transported to the laboratory and kept in a −20 °C freezer. The gelatin extraction method was performed with acetic acid (0.05 M) following the method of Abedinia et al. [16].

### 2.3. Preparation of Films

The bio-composite films were prepared based on the casting procedure described by Abedinia et al. [17]. Briefly, glycerol in a concentration of 25% (based on gelatin weight of 8 g) was weighed in a beaker containing 100 mL of distilled water. Subsequently, glycerol was dissolved and added to PG-FG blended powder to make 8% (*w*/*w*) 100:0, 75:25, 50:50, 25:75, and 0:100 film solutions, respectively. In order to hydrate the gelatin, the samples were stirred at room temperature for 30 min and then heated at 60 ± 2 °C to produce a clear solution (film solutions) and then the ultrasonic process (42 kHz, 135 W; Branson Ultrasonics, Brookfield, CT, USA) was used to remove the bubbles from the film solutions. The solutions were then cooled to 40 °C, and 45 g of the film solution was cast onto Perspex plates to give a film formation area of 16 × 16 cm^2^. The plates were dried in a humidity chamber under controlled conditions (25 ± 2 °C and 45 ± 5 percent relative humidity).

### 2.4. Characterization of Films

Before performing tests on the films, they were conditioned at 25 °C for 7 days in desiccators containing saturated solutions to achieve the desired relative RH (for mechanical, thickness, WVP and OP, at 50 ± 5% RH (Mg (NO_3_)_2_) and for WS, SR and FTIR, at 0% RH (P_2_O_5_)) [17].

#### 2.4.1. Film Thickness

Films thickness was measured with a hand-held micrometer (Thickness Gauge; Ozaki MFG Co., Tokyo, Japan) with eight replications. The thickness of each film was measured at eight different locations [18].

#### 2.4.2. Mechanical Properties

To determine the mechanical properties (TS, EB, and YM) of the films, strips of film 25 mm × 180 mm were measured by ASTM D882-18 [19] method using a texture analyzer (TA.XT2i Texture Analyzer; Stable Micro Systems, Godalming, Surrey) equipped with a 30 kg load. For each sample, the measurements were repeated five times, and the averages were considered as the results.

#### 2.4.3. Heat Seal Strength

Heat seal (HS) (N/m) value was determined following the standard test method (ASTM, E88-07a) [19] for seal strength of a flexible barrier material by applying a TA.XT2 texture analyzer equipped with Texture Exponent 32 software V.4.0.5.0 (SMS). A test speed of 90 mm/min and a 30-kg static load cell were used. Two strips (2.54 cm × 7.62 cm) of PG-FG films were placed on top of one another with a seal area of 0.2 × 2.54 cm^2^ and sealed with a semi-auto impulse sealer Model-SP-300H (Triumph Mercantile Corp, Taipei, Taiwan) for 3 s at 110 °C and heat seal pressure of approximately 1.8 × 105 Pa. The HS was obtained by dividing the maximum force required to peel off the seal (N) by the seal width (m).

#### 2.4.4. Light Transmittance and Color

The UV-Vis transmission ranges of films were measured at 200–800 nm by a UV-Vis spectrophotometer (model UV-160, Shimadzu, Kyoto, Japan) [20]. The well-controlled thickness film samples were placed in a spectrophotometer cuvette directly, and the air was considered as a reference.

The transparency is inversely proportional to the opacity of the film. The opacity values of films were calculated by Equation (2):

Transparency value = −Log T600/X(1)

where T600 is the value of transmittance at 600 nm, and X is the film thickness (mm). Absorbance values were also converted to transmittance values using the Lambert–Beer equation. To determine the appearance of films, three positions of each film surface were measured by a colorimeter (Minolta CM-3500D; Osaka, Japan). The obtained data were expressed on the as L*, a*, and b* scales and calibrated by a calibration plate (CM-A100) and air as full transmittance. Film specimens were placed on a black standard plate (L* = 1.35, a* = 0.03, and b* = −0.61). The total color difference (ΔE*) was calculated as Abedinia, Ariffin, Huda and Nafchi [17]:

ΔE* = [(L* − L)2 + (a* − a)2 + (b* − b)2] · 0.5(2)

where ΔL*, Δa* and Δb* are the differences between the corresponding color parameter of the samples and that of the white standard (L* = 93.58, a* = 0.88 and b* = 0.46).

#### 2.4.5. Attenuated Total Reflectance-Fourier Transform Infrared (ATR-FTIR) Analysis

In order to identify the functional groups and secondary structure of PG-FG films, the attenuated total reflectance-Fourier Transform Infrared Spectroscopy (ATR-FTIR) was performed by an infrared absorption spectrum. FTIR analysis of different concentrations of blended films was conducted as previously reported by Abedinia, Ariffin, Huda and Nafchi [16].

#### 2.4.6. Water Vapor Permeability (WVP)

The WVP of the samples was measured using the ASTM method E 96-95 [21]. The conditioned film samples were mounted on cups containing 25 mL of distilled water completely sealed. The cups were placed in a desiccator containing silica gel granules (as absorbents) at a temperature of 25 °C and a relative humidity of 0%. The weight of the cups was measured at intervals of 1 h and for a total of 12 h. Simple linear regression was used to estimate the slope of weight loss against the time graph.

WVP was calculated from the following formula:

WVP = (WVTR L)/Δp(3)

where WVTR (Water vapor transmission rate) is slope/film area [g/m^2^ × h], L is film thickness [mm], and Δp is partial water vapor pressure difference in kPa between the two sides of the film.

#### 2.4.7. Oxygen Permeability Assay (OP)

The OP values were measured performing the ASTM D 3985-05 [21] method. First, the oxygen transfer rate (OTR, cc/m^2^ day) through the PG-FG films was evaluated by an Automatic Gas Permeability tester (Minneapolis, MN, USA) at 23 ± 2 °C and 50% RH. The measurement was continued until a value became stable. The OP (cm^3^ µm m^−2^ day^−1^ kPa^−1^) was calculated by multiplying the OTR with film thickness (µm) and dividing with P (kPa).

### 2.5. Rheological Properties of Film Solutions

Using a temperature broad-range-controlled rheometer (AR1000-N, TA Instruments, Leatherhead, UK), the dynamic viscoelastic properties of film solutions were determined. The rheometer was equipped with a steel cone-plate geometry (cone angle = 2°, transition gap = 0.54 µm). A 6.67% (*w*/*v*) of each gelatin (FG, PG, PG-FG 50:50 and BG) in distilled water was heated and stirred at 50 °C for 30 min and was applied as a sample between the cone and plate of the rheometer. Silicon oil was placed over the outer edge of the sample to inhibit evaporation during frequency and time sweeps. The G′ is the storage or elastic modulus, which indicates the elastically stored energy amount in the structure, and the G′′ is the viscous or loss modulus, which indicates that the energy loss or the viscous response amount were identified as two rheological main parameters in this rheological test [16].

#### 2.5.1. Frequency Sweeps

A frequency sweep (applying a 319.7 Pa as stress value and changing the frequency from 0.1 rad/s to 100 rad/s) at 10 °C was performed according to the method described by Abedinia, Ariffin, Huda and Nafchi [16] to characterize the cross-linking behavior of gelatin gel (6.67%, *w*/*v*). All experiments were measured at the test temperature and in the detected linear viscoelastic region in three replications. Changes G′ and G′′ were recorded and determined as a function of frequency.

#### 2.5.2. Time Sweeps

A slightly time-dependent response to the stress used, with a storage modulus (G′), was shown in time sweep tests. This measurement was conducted at constant frequency and stress at 1 s^−1^ and 100 Pa, respectively, for 3 h at 10 °C. According to the preliminary results of the experiments, the optimal curing time was determined for all subsequent experiments [16].

### 2.6. Preparation of Sachets

The initial production and application of films as the packaging was carried out based on the study of Malherbi et al. [22], with some changes. In order to evaluate the stability of extra virgin olive oil from the films, packages in the form of rectangular bags (sachets) were made in dimensions of 6.5 × 3 cm (19.5 cm^2^), which are defined according to the commercial sachets’ dimensions, using the sealer and conditions used in the section of 2.3.3 for heat seal strength evaluation. The amount of 6 mL of olive oil was stored in bags and dried in a dryer (63 ± 3% RH). They were stored in a drying oven under accelerated oxidation conditions, simulating day and night at a temperature of 30 ± 3 °C, and the stability of the product was measured.

### 2.7. Stability Monitoring of Stored Oil

The bags or sachets containing oil were stored in a desiccator containing saturated sodium nitrate (63 ± 3% RH) inside an oven with a temperature of 30 ± 3 °C, considering day and night simulation. The acidity index (AI) and peroxide index (PI) of packaged extra virgin olive oil were measured according to the AOCS methodology [23] on days 0, 7 and 15 with three repetitions.

For AI determination, in brief, 2 g of the oil were placed into a container, followed by 20–30 mL of ethyl alcohol, 0.1 N NaOH reagent, and phenolphthalein indicator until the final solution was a stable shade of pink. The following equation was used to determine the results, and the results were expressed as a percentage of oleic acid:

AI% (Oleic acid/100 g sample) = (282 × N × V/100 × W)/100 (4)

where W denotes the sample’s weight used in this experiment, N denotes the normality of the sodium hydroxide (NaOH) used, V denotes the volume of the NaOH used, and 282 is the molecular weight of oleic acid.

For PI determination, a container was filled with roughly 2 g of the sample, 150 mL of the 3:2 acetic acid-chloroform mixture, and that was thoroughly mixed. The resultant solution was then added, thoroughly blended, and kept in the dark for one minute. Next, 0.5 mL of saturated potassium iodide was added. After that, 0.1 M sodium thiosulfate was added to the mixture and vigorously agitated until the final solution’s color changed to yellow. Then, 0.5 mL of the starch indicator was added, and the sodium thiosulfate titration was continued until the starch indicator’s blue color vanished. According to the following equation, the peroxide value was calculated, and the resulting value was expressed in mequiv (of active oxygen) per Kg^−1^ of the oil:

Peroxide valuve = (S × M)/G × 100 (5)

where S represents the utilized sodium thiosulfate’s volume (in mL), M is the used sodium thiosulfate’s molarity, and g is the sample’s weight (g).

### 2.8. Statistical Analysis

All experiments were run in triplicate with three different lots of films. One-way analysis of variance (ANOVA) and Duncan’s multiple range test using an SPSS package (SPSS for windows, SPSS Inc., Chicago, IL, USA) was used for statistical analyses. The statistically significant level was set at *p* < 0.05.

## 3. Results

### 3.1. Mechanical and Heat Seal Strength Properties of Blend Films

As shown in Table 1, the average thickness of the produced films shows that there is no significant difference between the samples (*p* > 0.05), which is due to the same nature of the two compounds in fabricating the films, both of which are gelatin in nature. The thickness of all films was between 0.114 and 0.125 mm. Film thickness did not change with increasing concentration or decreasing the ratio of gelatins. Typically, the mechanical properties of hydrocolloid films are studied based on three parameters: tensile strength (TS), Young’s modulus (YM), and percentage elongation at break (EB). Table 1 also shows the amount of TS, EAB, and Young’s modulus of PG/FG films that are combined with PG and FG at different levels. In general, all films incorporated with PG had higher TS (*p* < 0.05) than the control (FG), indicating that the interaction between protein chains was more pronounced, resulting in a stronger film network. The TS corresponds to the measured force (N) required to rupture the section of the films. The EB% is the ratio of displacement to the length of the reference sample. It is reported as a percentage relative to the comparison flexibility of films. The YM (MPa) is a parameter corresponding to the slope in the linear stress–strain curve for low elongations [24].

When PG concentration was increased from 0% to 100%, the hardness of the bio-composite film films hardness increased from 1.27 ± 0.32 to 17.03 ± 1.10 MPa and Young’s modulus increased from 25.72 ± 4 to 536.85 ± 74 MPa (*p* < 0.05). On the other hand, with increasing PG concentration, the percentage of elongation of the biocomposite films was reduced from 90.68 ± 4.26 to 29.14 ± 2.68 (*p* < 0.05). As a result, the mechanical properties of modified cold water fish gelatin bio-composite films were improved due to the gelatin matrix (FG) and fillers (PG) interaction.

Since, in food packaging, the heat seal strength of polymers plays a very prominent role in the stability of the stored food, selecting the most heat seal strength among the treatments considering the mechanical properties is of great importance [20]. The heat seal strength of the film samples increased with the increase of PG. The amount of this parameter showed a significant difference among all treatments (*p* < 0.05). The highest amount of heat seal strength was related to PG and BG film samples (232.31 and 238.15 N/m) and the lowest amount was related to FG film (62.19 N/m). However, in the samples where the amount of FG was higher, the amounts of EB and TS were not suitable for choosing them as a suitable film for use in making sachets. According to the measurements and visual evaluations of the samples, the 50:50 sample was chosen as the best sample to continue the production of sachets. Nilsuwan, Arnold, Benjakul, Prodpran and de la Caba [20], Malherbi, Schmitz, Grando, Bilck, Yamashita, Tormen, Fakhouri, Velasco and Bertan [25] and Abedinia, Ariffin, Huda and Nafchi [17] studies also confirm these results.

### 3.2. Barrier Properties of Films

#### 3.2.1. Water Vapor Permeability (WVP)

Cold water fish gelatin films have lower permeability values than MG and PG films [2,26]. The effects of PG on both permeabilities to water vapor and oxygen are presented in Table 2. The WVP value significantly increased with the addition of PG. Although this result has increased water vapor penetration to some extent, this amount is still far less than bovine and poultry gelatin films. Considering the favorable mechanical properties that adding PG gives to fish gelatin films, this increase can be ignored. It is suggested that PG increased free volume in these films, resulting in higher WVP. This is associated with the amino acids remaining in gelatins. In cold water fish gelatin, the increase in hydrophobicity is due to the decrease in proline and hydroxyproline amounts in its amino acid composition, so this amount is much higher in MG and PG [2]. The FG films can be particularly useful for applications related to reducing water loss from encapsulated drugs and frozen or refrigerated food systems due to its low water vapor permeability [18]. No significant difference (*p* > 0.05) was observed between the amount of WVP of PG and BG films, but a significant difference was observed with the addition of PG to FG. The highest WVP was observed in BG samples, and the lowest amount was observed in FG samples for 5.05 and 1.21 × 10^−11^ [g m^−1^ s^−1^ Pa^−1^], respectively. The results obtained by Parsaei, Mohammadi Nafchi, Nouri and Al-Hassan [18] and Abedinia, Ariffin, Huda and Nafchi [17] can justify these results.

#### 3.2.2. Oxygen Permeability (OP)

The results of oxygen permeability in Table 2 show that, with increasing concentration of PG in FG film, oxygen permeability also significantly increased (*p* < 0.05). The FG film had the lowest OP and the PG film had the highest OP. The chemical nature of macromolecules, the aggregation of molecules, and the degree of cross-linking are among the most important factors that affect the permeability of oxygen layers. The amount of certain amino acids in the protein can disrupt the formation of α-helix and affect the dynamic properties of gelatin. Proline and hydroxyl proline increase the strength of the α-helix structure by forming hydrogen bonds. Fish gelatins show a low OP due to their low content of proline and hydroxyl proline. The results of Parsaei, Mohammadi Nafchi, Nouri and Al-Hassan [18] and Nilsuwan et al. [27] confirm that, if cross-linking is created by chemical or natural compounds in the structure of gelatin, it leads to the reduction of OP.

### 3.3. Appearance and Light Barrier

**Figure 1:**
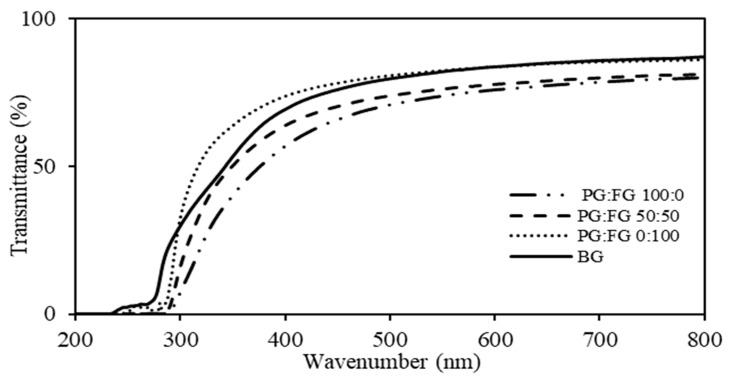
UV-Vis light spectrum of PG-FG gelatin biocomposite films.

**Figure 2:**
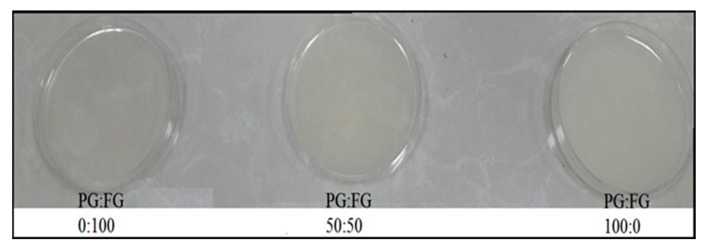
The appearance of the PG and FG films.

The UV-Vis light transmission spectrum of PG-FG gelatin biocomposite films measured in the wavelength range of 200 to 800 nm is shown in Figure 1. The light transmission of fish gelatin films after mixing with PG gelatin was significantly reduced and the graph shows that the reduction of light transmission was dependent on the concentration of PG. The light transmission of fish gelatin films after mixing with PG gelatin was significantly reduced, and the graph shows that the reduction of light transmission was dependent on the concentration of PG. In the UV light range, the light transmittance of the PG-containing biocomposite film is significantly reduced compared to the fish gelatin film, which indicates that the PG-FG biocomposite films have strong UV-blocking properties. It is noteworthy that UV light is almost completely blocked in the films for almost all PG weight ratios. As measures of UV light barrier and transparency properties, the percentage values of transmittance of the films at 600 nm (T600) are shown in Table 3, and Figure 2 has shown that, by adding PG to FG, the films become somewhat more opaque.

The PG-FG film transparency values were in the range of 14.77–15.53 (Table 3). By adding PG to FG, the transparency of the samples decreased. The highest transparency was related to the control sample (FG 100) with a rate of 14.77 and the most opaque film was PG-FG 50-50. No significant difference was observed between the transparency sample of bovine gelatin film, PG-FG 50-50, and PG100, but all films had a significant difference with the control sample (*p* < 0.05).

The PG-FG color characteristic parameters are summarized in Table 3. The lightness (L*) and parameter of a* significantly decreased, and significant increases were observed in b* and ΔE* with the increase of PG. These results represent the change of the film color to darker, greener and yellower. The difference between all values of color parameters in all samples was significant (*p* < 0.05). The yellowest film PG100 had the highest b* value equal to 3.9, and the bluest film was related to FG100 film with the b* value of 1.21. In addition, the greenest film PG100 with a value of parameter a* equal to −0.46, and the reddest film among the samples, was related to the control sample FG100 with a* value parameter equal to 0.13. In terms of chemical structure, proteins are heteropolymers of α-amino acids that differ in side groups. Therefore, their film response to pH changes during gelatin extraction is uncertain because they can act as buffer systems due to their ionizable side groups. In addition, amino acid side groups can be highly reactive to potential cross-linking or chemical bonding. On the other hand, due to the different compositions of amino acids in PG and FG, this color difference has been observed. Consequently, the levels of the aromatic amino acids tyrosine and phenylalanine in duck feet gelatin (DFG) were quite similar to those of bovine (18.41, 4.05, and 16.02 and 10.6 residues/1000 residues for DFG and bovine gelatin, respectively). In general, sensitive chromophores such as tyrosine and phenylalanine absorb light with a wavelength of less than 300 nm [16,17]. The results of Hazaveh, Mohammadi Nafchi and Abbaspour [26], and Nilsuwan, Arnold, Benjakul, Prodpran and de la Caba [20] are consistent with our results.

### 3.4. ATR-FTIR Evaluation

**Figure 3:**
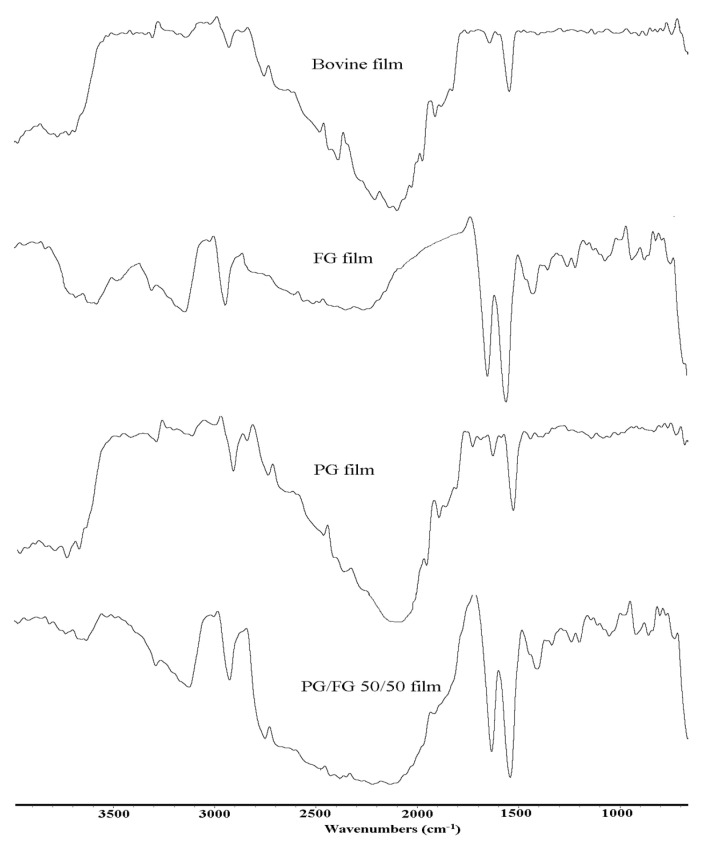
ATR−FTIR spectra of PG, FG, PG−FG 50-50, and BG gelatin films.

FTIR spectroscopy is a fast and non-destructive method. ATR-FTIR spectra of PG, FG, PG-FG 50-50 and BG gelatin films were measured in order to clarify the gelatin–gelatin interactions. In Figure 3 and Table 4, five main clear areas are observed. The spectra of films containing fish gelatin are different from PG and BG. Therefore, the changes in the secondary structure of the protein in the mixed gelatin are clearly revealed by comparing the FTIR spectra of the gelatins. In this study, the identified region includes 1644–1656 (Amide I), 1335–1560 (Amide II), 2300–3600 (Amide A), and 670–1240 cm^−1^ (Amide III); the amide A group was recorded at 3304, 3291, 3292, and 3302 cm^−1^ for PG-FG 100-0, PG-FG 50-50, PG-FG 0-100, and BG, respectively, which are close to the frequency of NH free stretch (N-H stretch coupled with H-bond). The lower peak indicates changes in the secondary structure of collagen [28]. The amide B band was recorded with a lower value in the samples with PG and the highest value in the FG sample, that is, 2923.74, 2926.15, 2927.71, and 2924.77 cm^−1^ for PG-FG 0-100, PG-FG 50-50, PG-FG 0-100, and BG, respectively, which is equivalent to CH antisymmetric and symmetric stretching. Amongst the gelatin films, FG showed the lowest peak amide B wave number, indicating the single bond NH3 group interactions between peptide chains. A shoulder peak is also seen in the PG100 film at 2856 cm^−1^, which indicates CH2 single bond CH3 stretching vibrations [29]. The carbonyl C=O double bond stretching mode dominates the amide I band, with contributions from the CN stretch, CCN deformation and in-plane NH bending modes and is mostly found in the region of 1660 cm^−1^ to 1620 cm^−1^, while this band in the samples at 1643.07 for PG 100, 1635.71 for PG-FG 50-50, 1633.71 for FG 100, and 1638.58 cm^−1^ for BG was observed. The lowest amount of amide I was from FG. The frequency range of 1640 cm^−1^ to 1620 cm^−1^ shows β-sheet structures [16]. In the amide II region, the peaks at 1542.07, 1541.96, 1541.83, and 1541.57 cm^−1^ were detectable for PG-FG 0-100, PG-FG 50-50, PG-FG 0- 100, and BG, respectively. The wavenumber range of 1550–1520 cm^−1^ is created by α-helix structure in the range of 1550–1540 cm^−1^ and β-sheets at 1525–1520 cm^−1^. The in-plane NH deformation and out-of-phase CN stretching modes of the peptide group are responsible for the vibrational modes of amide II. Although this band is typically thought to be more sensitive to water than to changes in protein conformation, its location also reveals changes in the secondary structure of gelatin [30]. The amplitude of amide II of the sample shows that, with the addition of PG to FG compared to FG and BG, this amplitude has decreased, which suggests that N-H was less active in forming bonds with nearby α chains. The amide III band was detected around the wave numbers 1220 to 1239 cm^−1^, indicating the combination of peaks between C single bond stretching vibrations and NHH vibrations. In the amide III region, PG and BG samples had lower amplitude than FG. The conversion of an alpha-helix to a random coil structure has occurred in connection with the type of gelatin and is caused by the deficiency of some helix-forming amino acids [16].

### 3.5. Rheology Evaluation of Film Solutions

#### 3.5.1. Effect of PG on Viscosity

**Figure 4:**
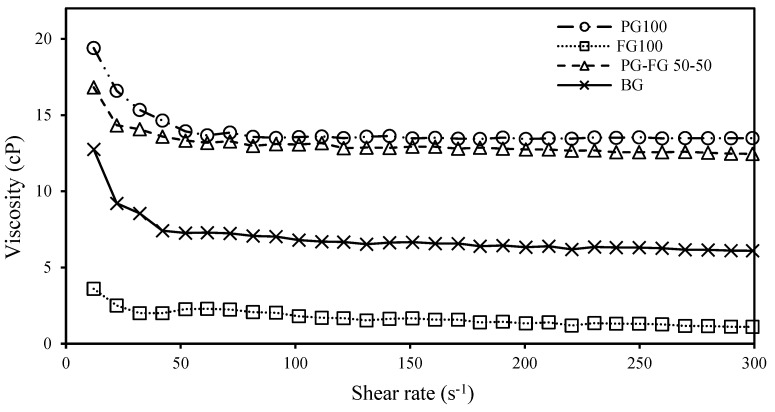
The viscosity of PG, FG, PG−FG and BG gelatin solution films.

Figure 4 shows the viscosity of gelatin solutions. In an overall view, the graph has no cuts, so the measurements do not become equal in any specific second. Pure FG has the least viscosity of all, and bovine has a higher viscosity, on a scale of nearly 5cP. The PG-FG 50-50 and PG100 both have a viscosity almost twice as much as bovine, with a little difference of FG being a little higher (about 10 cP higher than FG100), indicating that the gelatin of poultry and fish can achieve good viscosity. In addition, the PG’s viscosity at the end of the graph becomes almost equal to the bovine’s initial. The PG100 and PG−FG’s difference in viscosity is the highest at first and least in between (50–100 s), and then it starts to become slightly more till the end. All gelatins have a fall of viscosity measurement at first, and then the rate is almost constant. For bovine, we can see the greatest fall in the initial 10 s and the least for FG100. As we can see, the FG100’s viscosity is initially 3.6 cP, but when mixed with the PG, it reaches a point of 16.8 cP. After about 300 s, the values of 1.1, 6.1, 12.46 and 13.49 cP for FG, bovine, PG−FG, and PG were observed, respectively. The viscosity is a measure of a fluid’s internal resistance to flow and shear under the force of gravity. In the case of gelatin, it is determined by molecular weight as well as the polydispersity of the gelatin polypeptides or amino acids [31]. The higher the gelatin gel strength, the higher the viscosity, which is attributed to the higher proportion of cross-linking components (ß- and Ƴ-components) [16].

#### 3.5.2. Effect of PG on Frequency Sweeps

**Figure 5:**
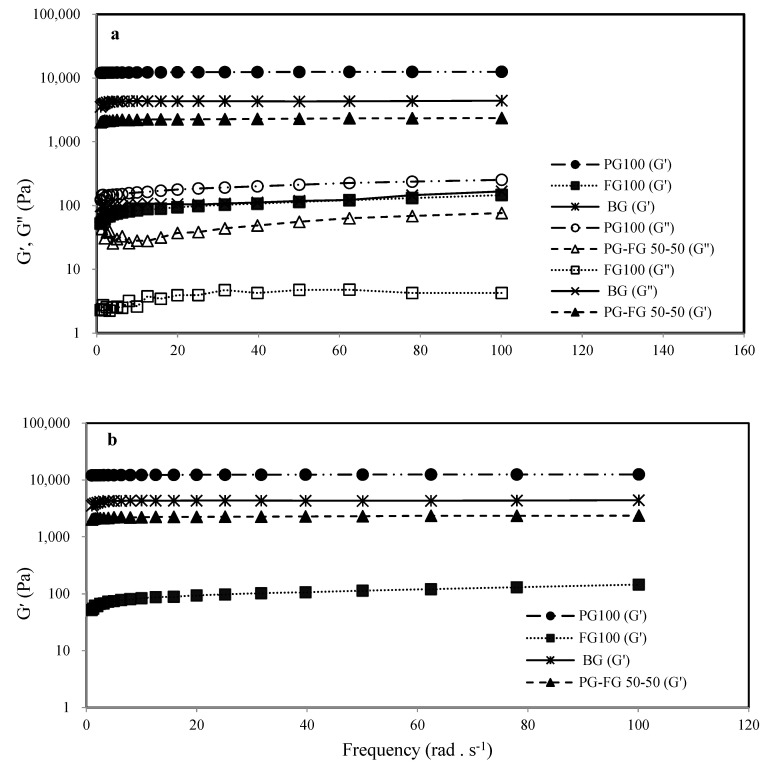

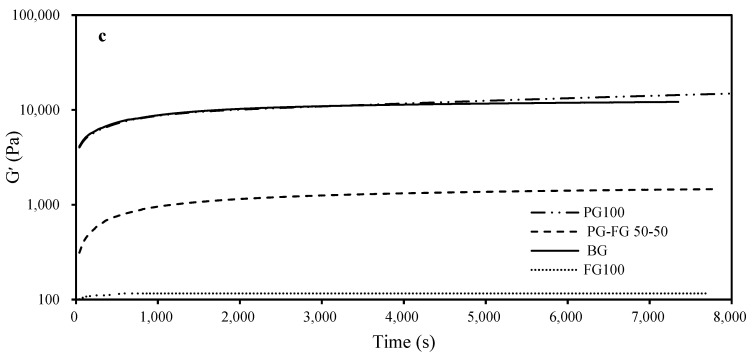
Rheological parameters at constant temperature (10 °C) using 6.67% (*w*/*w*) solutions of gelatin samples; (**a**) and (**b**) frequency sweeps; G′ and G′ as a function of frequency over 0–100 rad/s for 6.67% (*w*/*w*) gelatin sample solutions and (**c**) time sweep.

To evaluate the strength of the gel network, we can use the frequency dependence study of G′. It is used to provide a significant description of the rheological behavior of the product during storage and use. For this purpose, in this study, by utilizing 6.67% (*w*/*w*) solutions of gelatin samples in a dynamic rheological test (as a function of frequency over 0–100 rad/s) at constant temperature (10 °C), the crosslinking behavior of PG-FG gels was assessed. Figure 5a,b demonstrate the frequency dependence of G′ and G′, G′′ for sample solutions. The G′ values are greater than G′′. All solutions containing PG and BG reached their gelation point at 10 °C, which led to network formation and high G′ values. A linear increase in G′ with frequency indicates a stable gel network in a specific frequency range. However, the FG solution shows very low values of G′ and has not created a stable network. The graphs show that the addition of PG has a direct positive effect on the strength of the FG network. The G and G′′ values for PG100, BG and PG-FG 50-50 were independent of frequency and greater than those of FG because samples containing PG and BG produced a stronger gel than samples containing FG. This outcome also demonstrated that FG exhibits poorer intermolecular interactions when compared to samples that contain PG and BG. All gelatin gels were essentially frequency-independent. This observation was in agreement with the study of Cen, Zhang, Liu, Lou, Wang and Huang [14]. The percentage of G′ protein rises when a significant amount of energy from the deformed material is elastically stored in the gel network by increasing the number of intermolecular crosslinks, resulting in a more cohesive matrix [2].

#### 3.5.3. Effect of PG on Time Sweep

Figure 5c demonstrates that some samples’ responses to applied stress are slightly time-dependent, and G′ gradually declines with time. The greatest and very identical values of G′ were found in the PG and BG gelatin solutions. Of course, the graph shows that the values of G′ for BG compared to PG have relatively decreased with increasing time at the end. A little lower than the above curves, the 50-50 PG-FG curve is located, which was still increasing over time. The lowest G′ was given by the FG curve, which was considerably below the value of 10 Pa. This result demonstrated the strength of gels containing PG and was in good agreement with other rheological findings.

### 3.6. Evaluation of the Stability of Olive Oil in Sachets

**Figure 6:**
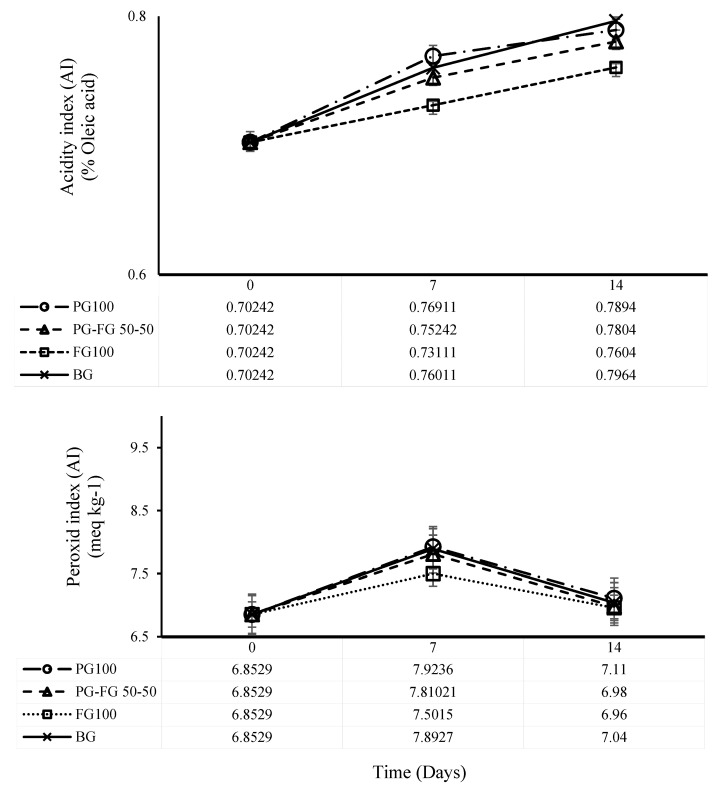
Acidity index (AI) and peroxide index (PI) of olive oil during 14 days of storage at room temperature in PG-FG-based sachet films.

Acidity index (AI) and peroxide index (PI) were evaluated during 14 days of storage at room temperature. As shown in the results of Figure 6, the average values of the AI (% oleic acid), regardless of the type of film used for packaging, were not significantly different (*p* > 0.05) in relation to the storage period. In this study, it was observed that, in all three films used for packaging as sachets and controls, the quality of extra virgin olive oil was within the range determined by WHO [32] (<0.8%) until the end of the storage period. Malherbi, Schmitz, Grando, Bilck, Yamashita, Tormen, Fakhouri, Velasco and Bertan [25] achieved results close to those presented in this study when studying corn starch and gelatin-based films with guabiroba pulp for application in food packaging during a 14-day storage period.

The effect of incorporation of PG into FG in bags during the 15-day storage period showed that the values obtained for peroxide index or PI showed values from 6.85 to about 7.9 (meq kg^−1^) after 7 days in both PG100 and BG films. It found that a statistically significant difference (*p* < 0.05) increased between treatments. It is probably due to the presence of oxygen that remained inside the package during the packaging process in the headspace section, causing the oxidation of lipids in the first days of product storage [33], which can be seen in the results of the 7th day. No statistical difference (*p* > 0.05) was observed in PI value from day 7 to 15 for any of the films during the study period. Some studies have reported results similar to those here. For example, Carpiné et al. [34] observed that olive oil stored in biodegradable layers of soy protein isolate, coconut oil and natural surfactants experienced an increase in PI from 7.59 to 13.197 kg^−1^ in 28 days. Malherbi, Schmitz, Grando, Bilck, Yamashita, Tormen, Fakhouri, Velasco and Bertan [25] observed that, after the 7th day, the values of PI increased from 6.14 to 8.21 meq kg^−1^. It confirms that the stability of packaged olive oil was visible under the influence of time for more than 14 days.

**Table 4:** FTIR evaluation of poultry gelatin-fish gelatin blended films.

Region	Peak Wave Number (cm^−1^)				Assignment	Reference
	PG-FG 100:0	PG-FG 50:50	PG-FG 0:100	BG-		
Amide A	3304	3291	3292	3302	N-H stretch coupled with H-bond	[17]
Amide B	2923	2927	2927	2924	CH antisymmetric and symmetric stretching	[35]
	2923	2927	2927	2924	CH2 asymmetrical stretching	[36]
Amide Ι	1643	1634	1633	1638	C=O stretch/hydrogen bond coupled with COO-	[17]
Amide ΙΙ	1542	1543	1541	1541	NH bend coupled with CN stretch	[37]
Amide ΙΙΙ	1219	1200	1201	-	NH bend stretch coupled CN stretch	[37]
Fingerprint	1097	1053	1054	1002	C–O skeletal stretch	[37]

## 4. Conclusions

The production of biodegradable and edible films that ensure the possibility of using them easily is still being studied. In this application, the use of alternative MG (PG and FG) was blended to synergize their properties. They were able to successfully enhance each other’s better qualities in the produced film. Having the favorable characteristics of film formation of FG, such as a low amount of WVP compared to other gelatin films and, on the other hand, by poultry gelatin, which has the favorable characteristics of film formation and the favorable structure of the gel, the results showed that the combination of 50-50 of these gelatins can form a film that can be used as a virgin olive oil package for 14 days without a drop in acidity and peroxide, which makes it suitable for industrial food applications.

## Figures and Tables

**Table 1:** Thickness (T), mechanical and heat seal strength (HS) of films.

PG-FG (Ratio)	T (mm)	TS (MPa)	EB (%)	YM (MPa)	HS (N/m)
100:0	0.125 ± 0.02 ^a^	17.03 ± 1.10 ^b^	29.14 ± 2.68 ^f^	536.85 ± 74 ^a^	232.31 ± 10.21 ^a^
75:25	0.121 ± 0.03 ^a^	10.96 ± 1.63 ^c^	36.10 ± 3.63 ^e^	247.25 ± 33 ^c^	183.15 ± 14.09 ^b^
50:50	0.114 ± 0.01 ^a^	7.01 ± 0.29 ^d^	62.22 ± 2.34 ^c^	111.41 ± 39 ^d^	152.21 ± 15.32 ^c^
25:75	0.122 ± 0.04 ^a^	3.11 ± 0.17 ^e^	73.15 ± 2.62 ^b^	47.38 ± 11 ^e^	110.22 ± 18.14 ^d^
0:100	0.118 ± 0.02 ^a^	1.27 ± 0.32 ^f^	90.68 ± 4.26 ^a^	25.72 ± 4 ^f^	62.19 ± 28.62 ^e^
BG	0.123 ± 0.04 ^a^	18.68 ± 0.17 ^a^	48.97 ± 3.04 ^d^	382.81 ± 39 ^b^	238.15 ± 11.03 ^a^

Amounts are presented as mean of five replications ± standard deviation. Different superscripts in the same column indicate significant differences (*p* < 0.05).

**Table 2:** Effects of poultry gelatin on permeability properties of cold water fish gelatin films.

PG-FG (Ratio)	WVP × 10^−11^ [g m^−1^ s^−1^ Pa^−1^]	O.P [cm^3^ μm/(m^2^ day)]
100:0	4.95 ± 0.28 ^a^	97.46 ± 1.06 ^a^
50:50	2.68 ± 0.18 ^b^	60.36 ± 1.01 ^c^
0:100	1.21 ± 0.12 ^c^	48.13 ± 1.13 ^d^
BG	5.05 ± 0.04 ^a^	80.40 ± 1.62 ^b^

The mean of three replications ± standard deviation is given as result. Different superscripts in the same column indicate significant differences (*p* < 0.05).

**Table 3:** Color properties and transparency of poultry gelatin-fish gelatin blended films.

PG-FG (Ratio)	L*	a*	b*	ΔE*	Transparency
100:0	95.28 ± 0.28 ^d^	−0.46 ± 0.006 ^c^	3.90 ± 0.09 ^b^	4.07 ± 0.12 ^b^	15.19 ± 1.04 ^a^
50:50	95.77 ± 0.18 ^b^	−0.36 ± 0.0001 ^b^	2.87 ± 0.002 ^c^	3.49 ± 0.08 ^c^	15.53 ± 1.03 ^a^
0:100	96.34 ± 0.12 ^a^	0.13 ± 0.003 ^a^	1.21 ± 0.04 ^d^	2.96 ± 0.09 ^d^	14.77 ± 1.09 ^b^
BG	95.57 ± 0.004 ^c^	−0.40 ± 0.002 ^d^	4.07 ± 0.01 ^a^	4.32 ± 0.11 ^a^	15.37 ± 1.01 ^a^

Values are given as mean ± SD (*n* = 3). Different letters within the same column indicate significant differences (*p* < 0.05).

## Data Availability

The data that support the findings of this study are available from the corresponding authors upon reasonable request.
